# In Vitro Antibacterial and Anti-Inflammatory Activity of *Arctostaphylos uva-ursi* Leaf Extract against *Cutibacterium acnes*

**DOI:** 10.3390/pharmaceutics14091952

**Published:** 2022-09-15

**Authors:** Federica Dell’Annunziata, Stefania Cometa, Roberta Della Marca, Francesco Busto, Veronica Folliero, Gianluigi Franci, Massimiliano Galdiero, Elvira De Giglio, Anna De Filippis

**Affiliations:** 1 Department of Experimental Medicine, University of Campania “Luigi Vanvitelli”, 80138 Naples, Italy; 2 Jaber Innovation s.r.l., Via Calcutta 8, 00144 Rome, Italy; 3 Department of Chemistry, University of Bari, Via Orabona 4, 70126 Bari, Italy; 4 Department of Medicine, Surgery and Dentistry “Scuola Medica Salernitana”, University of Salerno, 84081 Baronissi, Italy; 5 Clinical Pathology and Microbiology Unit, San Giovanni di Dio e Ruggi D’Aragona University Hospital, 84126 Salerno, Italy; 6 NSTM, National Consortium of Materials Science and Technology, Via G. Giusti 9, 50121 Florence, Italy

**Keywords:** *Cutibacterium acnes*, *Arctostaphylos uva-ursi* leaf extract, antimicrobial activity, biofilm degradation, anti-inflammatory efficacy

## Abstract

*Cutibacterium acnes* (*C. acnes*) is the main causative agent of acne vulgaris. The study aims to evaluate the antimicrobial activity of a natural product, *Arctostaphylos uva-ursi* leaf extract, against *C. acnes*. Preliminary chemical–physical characterization of the extract was carried out by means of FT-IR, TGA and XPS analyses. Skin permeation kinetics of the extract conveyed by a toning lotion was studied in vitro by Franz diffusion cell, monitoring the permeated arbutin (as the target component of the extract) and the total phenols by HPLC and UV-visible spectrophotometry, respectively. Antimicrobial activity and time-killing assays were performed to evaluate the effects of *Arctostaphylos uva-ursi* leaf extract against planktonic *C. acnes*. The influence of different *Arctostaphylos uva-ursi* leaf extract concentrations on the biofilm biomass inhibition and degradation was evaluated by the crystal violet (CV) method. The 3-(4,5-dimethylthiazol-2-yl)-2,5-diphenyl tetrazolium bromide (MTT) test was used to determine the viability of immortalized human keratinocytes (HaCaT) after exposure to *Arctostaphylos uva-ursi* leaf extract for 24 and 48 h. Levels of interleukin (IL)-1β, IL-6, IL-8 and tumour necrosis factor (TNF)-α were quantified after HaCaT cells cotreatment with *Arctostaphylos uva-ursi* leaf extract and heat-killed *C. acnes*. The minimum inhibitory concentration (MIC) which exerted a bacteriostatic action on 90% of planktonic *C. acnes* (MIC_90_) was 0.6 mg/mL. Furthermore, MIC and sub-MIC concentrations influenced the biofilm formation phases, recording a percentage of inhibition that exceeded 50 and 40% at 0.6 and 0.3 mg/mL. *Arctostaphylos uva-ursi* leaf extract disrupted biofilm biomass of 57 and 45% at the same concentrations mentioned above. Active *Arctostaphylos uva-ursi* leaf extract doses did not affect the viability of HaCaT cells. On the other hand, at 1.25 and 0.6 mg/mL, complete inhibition of the secretion of pro-inflammatory cytokines was recorded. Taken together, these results indicate that *Arctostaphylos uva-ursi* leaf extract could represent a natural product to counter the virulence of *C. acnes,* representing a new alternative therapeutic option for the treatment of acne vulgaris.

## 1. Introduction

Acne vulgaris is a multifactorial chronic inflammatory dermatosis of the pilosebaceous follicles, mainly manifested in adolescents [1]. Although it is not a life-threatening disease, it has a great social and psychological impact on the patient’s life [2]. Furthermore, it is estimated to be the eighth most common disease in the world, with 650 million people affected [3]. The onset of the disease occurs due to obstruction of the hair follicular ducts, preventing the release of sebum from the sebaceous glands and the formation of inflammatory lesions (papules, pustules and nodules) and comedones [4,5]. This condition determines the occurrence of primary acne, which mainly affects the skin of the face, neck and upper torso [6]. The prevalence of the disease varies according to age and sex. It is estimated that about 40% of women aged 14–17 and 37% of males aged 16–19 manifest pimples [7]. However, there are documented cases of acne in children younger than 9 years [8].

The main bacteria colonizing the sebaceous glands and responsible for the onset of acne vulgaris are *Staphylococcus epidermidis* and *Cutibacterium acnes* (*C. acnes*) [9]. These species are cutaneous commensals but in altered skin conditions become invasive [10]. Their pathogenicity derives from the production of extracellular lipase which hydrolyzes sebum triglycerides into glycerol and free fatty acids, which contribute to follicular damage [11]. Furthermore, they promote the inflammatory response by stimulating monocytes to secrete pro-inflammatory cytokines, including interleukin (IL)-1β, IL-8 and tumour necrosis factor (TNF)-α [12].

The conventional antibiotics for the treatment of this disorder are for systemic or topical use [13,14]. The first, reserved for severe acne, include tetracyclines; for topical use clindamycin, tetracycline and erythromycin are commonly used [15,16]. However, prolonged and improper use of antibiotics leads to numerous side effects (skin irritation, redness, skin irritation and hyperpigmentation) and the development of resistant multidrug microorganisms [17,18,19]. Therefore, the American Academy of Dermatology recommends the use of antibiotics limited to the shortest possible times and with a re-evaluation of the drug every 3–4 months to minimize the onset of resistance [20]. These limitations have underscored the need to identify new, safe, effective and low-cost acne treatment strategies [21]. Contextually, the use of natural substrates has aroused considerable interest in the scientific community for several years [22,23].

Natural products represent a great source of bioactive compounds and have numerous advantages: (i) minimal side effects in the patient; (ii) use of renewable compounds; (iii) low production costs compared to synthetic drugs; and iv) reduced propensity to develop drug resistance [24]. Widely used in ethnopharmacology are the Ericaceae plants [25,26]. This family is represented, for example, by the bearberry (*Arctostaphylos uva-ursi* (L.) Spreng), an evergreen dwarf shrub that colonizes the alpine regions of Europe, Asia and North America [27]. The bearberry leaves are widely used as a diuretic and anti-inflammatory agent to counteract several urogenital tract diseases [28]. Leaf extracts have also been proposed as a natural antioxidant additive due to the high content of phenolic compounds [29]. The latter, present in the raw leaf extract, also showed antiproliferative properties against five carcinoma cell lines, MCF-7 (estrogen receptor-positive breast cancer), DU-145 (androgen receptor-negative prostate cancer), HT-29 (colon cancer), SK-MEL-5 and MDA-MB-435 (melanoma; skin cancer) [30].

Current knowledge of the beneficial properties associated with bearberry laid the foundation for the investigation of the *Arctostaphylos uva-ursi* leaf extract as an antimicrobial agent. The effective antibacterial activity associated with a powerful anti-inflammatory action suggested that *Arctostaphylos uva-ursi* could represent a promising source for the treatment of acne vulgaris.

The purpose of this work is a detailed analytical characterization of the extract by different techniques: Fourier Transform Infrared Spectroscopy (FT-IR) in Attenuated Total Reflectance (ATR), X-ray Photoelectron Spectroscopy (XPS) and Thermo-Gravimetric Analysis (TGA). The total phenol content (TPC) was determined by the Folin–Ciocalteu colourimetric method, while a target component (i.e., arbutin) was quantified by High-Performance Liquid Chromatography (HPLC). Radical scavenging activity was in vitro investigated by DPPH assay. Moreover, Franz diffusion cell was employed to ascertain the in vitro permeation capabilities of a toning lotion loaded with *Arctostaphylos uva-ursi* leaf extract, monitoring arbutin and TPC in the receptor compartment. Finally, the evaluation of antibacterial activity associated with a powerful anti-inflammatory action suggests the use of *Arctostaphylos uva-ursi* could as a promising source for the treatment of acne vulgaris.

## 2. Materials and Methods

### 2.1. Bacterial Strain, Compounds and Materials

*C. acnes* ATCC 11827 was purchased from the American Type Culture Collection (ATCC, Manassas, Virginia). For bacterial growth, Schaedler Anaerobe broth (casein enzymic hydrolysate 5.67 g/L, L-cystine 0.40 g/L, dextrose 5.83 g/L, dipotassium hydrogen phosphate 0.83 g/L, hemin 0.010 g/L, papaic digest of soybean meal 1.0 g/L, proteose peptone 5.0 g/L, sodium chloride 1.67 g/L, tris hydroxymethyl aminomethane 3.0 g/L, yeast extract 5.0 g/L) (Condalab, Madrid, Spanish), Schaedler Anaerobe Agar (Oxoid, Thermo Fischer Scientific, Lowell, MA, USA) and Fastidious Anaerobe Agar (FAA-agar) (peptone Mix 23.0 g/L, sodium chloride 5.0 g/L, soluble starch 1.0 g/L, sodium bicarbonate 0.4 g/L, glucose 1.0 g/L, sodium pyruvate 1.0 g/L, cysteine HCl monohydrate 0.5 g/L, hemin 0.01 g/L, vitamin K 0.001 g/L, L-arginine 1.0 g/L, soluble pyrophosphate 0.25 g/L, sodium succinate 0.5 g/L) (Thermo Fischer Scientific, MA, USA) were used [31,32]. Phosphate Buffered Saline 1X (PBS 1X) (sodium chloride 8 g/L, potassium chloride 0.2 g/L, dibasic sodium phosphate 1.44 g/L, monobasic potassium phosphate 0.24 g/L) (Sigma-Aldrich, Saint Louis, MO, USA) was used to dilute and/or wash bacteria. *Arctostaphylos uva-ursi* leaf extract, total hydroquinone derivatives expressed as anhydrous arbutin in the range 19.0–21.0 w/w, was purchased from Farmalabor (Canosa di Puglia, Italy). Arbutin standard for HPLC analysis, gallic acid (GA), 1,1-diphenyl-2-picrylhydrazyl (DPPH), sodium carbonate and Folin–Ciocalteu’s phenol reagent were supplied by Sigma Aldrich. The compound was dissolved in sterile ultrapure water (Sigma-Aldrich, MO, USA) at the final concentration of 20 mg/mL and stored at −20 °C until use.

### 2.2. Characterization of Arctostaphylos uva-ursi Extract

#### 2.2.1. FT-IR/ATR Analysis

FT-IR/ATR analysis was performed through a Spectrum Two PE instrument supplied by PerkinElmer, endowed with a universal ATR accessory (UATR, Single Reflection Diamond/ZnSe). For each sample, FT-IR/ATR spectra were recorded from 400 to 4000 cm-1 with a 4 cm-1 resolution.

#### 2.2.2. Thermal Analysis

Thermogravimetric analysis (TGA) of *Arctostaphylos uva-ursi extract* was carried out using the PerkinElmer TGA-400 analyzer (PerkinElmer Inc., Waltham, MA, USA). Samples of approximately 5–10 mg were placed in a ceramic crucible and heated under N_2_ atmosphere from 30 to 800 °C, with a constant flow rate of 20 °C/min and at a gas flow of 20 mL/min. Thermograms (TG) and their respective derivative curves (DTG) were recorded and analyzed by TGA Pyris series software (version 13.3.1.0014, PerkinElmer Inc., Waltham, MA, USA).

#### 2.2.3. XPS Characterization

XPS characterization was carried out using a scanning microprobe PHI 5000 VersaProbe II, purchased from Physical Electronics (Chanhassen, MND, USA), equipped with a micro-focused monochromatized AlKα X-ray source. Specimens were analyzed in HP mode using an X-ray take-off angle of 45°. The instrument base pressure was set at about 10^−9^ mbar. Scanned areas of 1400 × 200 μm were acquired. Pass energy values equal to 117.4 and 29.35 eV were recorded in the FAT mode for survey scans and high-resolution spectra, respectively. For high-resolution spectra curve fitting, MultiPak software (version 9.9.0.8, Physical Electronics, Chanhassen, MN, USA) was used. The carbon C1s correction was performed by setting as reference charge (284.8 eV) the adventitious carbon.

#### 2.2.4. Total Polyphenol Content (TPC) Determination

TPC present in the *Arctostaphylos uva-ursi* extract was determined in triplicate by the Folin–Ciocalteu spectrophotometric method, described by Fabiano and coworkers, with some modifications [33]. Briefly, for the calibration curve, gallic acid (GA) stock solution at a concentration 5 g/L was prepared by dissolving 0.5 g of GA in 10 mL ethanol and then bringing to 100 mL with distilled water. Successively, dilutions of this stock solution were performed to obtain different concentrations of GA (i.e., 100, 150, 250, 500 mg/L). A 100 μL aliquot of each GA solution was added to 7.9 mL of H_2_O and 500 µL of Folin–Ciocalteu reagent. The solution was left for 8 min before being added with 1.5 mL of sodium carbonate solution (1.9 M). The resulting absorbance was measured spectrophotometrically using a UV-visible Spectrophotometer UV-1900i (Shimadzu, Kyoto, Japan) against the blank at 765 nm. The TPC was calculated by referring to the calibration curve and expressed as mg of gallic acid equivalent per gram of dry extract (GAE/g).

#### 2.2.5. Preparation of a Toning Lotion Loaded with *Arctostaphylos uva-ursi* Leaf Extract

Toning lotions are water-based formulations usually containing water-soluble active ingredients, used to remove impurities (pollutants, makeup residues, sebum, etc.), refresh the skin and balance the natural pH level. When precious botanical extracts are contained in toning lotions, their action is mainly linked to the extract beneficial properties (astringent, moisturizing, antiaging action, etc.). In this study, an alcohol-free toning lotion formulation was produced, employing a simple recipe based on the solubilization of *Arctostaphylos uva-ursi* leaf extract in distilled water in a non-cytotoxic and antibacterial effective dose (i.e., 1.2 mg/mL). Finally, an eco-certified preservative (i.e., a mixture of benzyl alcohol and dehydroacetic acid, supplied by Zenstore, Salerno, Italy) was added to the lotion at 0.6% *w/w*.

#### 2.2.6. HPLC Analysis

HPLC (Prominence Series 20 with SPD-M20A PDA detector, Shimadzu, Milan, Italy) analysis of *Arctostaphylos uva-ursi* extract was carried out using arbutin as the reference compound, as previously described [34]. Samples were tested in triplicate, and results were reported as mean ± standard deviation. The analytical column used was a Shim-Pack GIST C18-AQ column (150 mm × 4.6 mm, 5 µm Shimadzu) with a mobile phase consisting of solvent A (85%water) and B (15% methanol), with a flow rate of 1 mL/min, using an isocratic mode elution at 30 °C and monitoring the effluent at 282 nm. The samples injection was made through a 20 µL injection loop. The calibration curve was built up by weighing appropriate amounts of the reference compound dissolved in a mobile phase to obtain the four desired concentrations (i.e., 1, 5, 25, 100 µg/mL). Finally, results were elaborated by using the LabSolutions Lite for LC-PDA software (version 5.82, Shimadzu Corporation, Milan, Italy).

#### 2.2.7. In Vitro Skin Permeation Studies

In vitro skin permeation experiments of Arctostaphylos uva-ursi leaf extract were carried out using a jacketed Franz diffusion cell (PermeGear Inc., SES GmbH, Bechenheim, Germany), as reported in a previous study [35]. In detail, 1 mL of toning solution containing the extract (1.2 mg/mL) was placed in the cell donor compartment in contact with the synthetic StratM^®^ membrane (Merck KGaA, Darmstadt, Germany). This membrane was chosen since it possessed a skin-like porosity, diffusivity and composition. An O-ring joint was put between the donor cell and the membrane, and the whole assembly was fixed with a stainless-steel clamp to guarantee the tight connection between the two compartments. The receptor compartment was filled with 5 mL of PBS (pH 7.4) and kept under continuous agitation by an ATE magnetic stirrer (VELP Scientifica Srl, Usmate, Italy). The temperature was fixed at 32.00 ± 0.03 °C using a CD-B5 heating circulator bath (Julabo GmbH, Seelbach, Germany). PBS aliquots of 500 µL were withdrawn after 2, 6 and 24 h, replaced with fresh PBS, and their arbutin content was assessed by HPLC (Prominence Series 20 with SPD-M20A PDA detector, Shimadzu, Milan, Italy), as described in the HPLC Analysis Section. Furthermore, at the end of the experiments, the Strat-M^®^ membrane was immersed in the HPLC mobile phase overnight at 25 °C in order to estimate the arbutin engaged in the membrane. At the same time points, total polyphenols permeated and retained by the membrane were also assayed, following the above-reported TPC procedure.

### 2.3. Kirby–Bauer Disk Diffusion Test

An initial screening to assess the *Arctostaphylos uva-ursi* leaf extract antimicrobial susceptibility against *C. acnes* ATCC 11827 was performed by the Kirby–Bauer disc diffusion test according to the guidelines of the National Committee on Clinical Laboratory Standards (NCCLS). In detail, a fresh colony was inoculated in a physiological solution up to a turbidity of 1 McFarland. The bacterial suspension was uniformly seeded with a sterile swab on FAA-agar. A blank disc placed on the FAA-agar plate was soaked in 1 mg of extract (UV), while a blank disc soaked in H_2_O was used as solvent control (S). Benzylpenicillin (1 μg) (Thermo Fisher Scientific, Waltham, MA, USA) was used as antibiotic control (PG). The plates were incubated at 37 °C for 48 h in an anaerobic environment. After incubation, the diameters of the areas of inhibition were measured.

### 2.4. Determination of the Minimum Inhibitory Concentration (MIC)

The *Arctostaphylos uva-ursi* leaf extract antibacterial activity was evaluated through the plate microdilution test according to the guidelines of the Clinical and Laboratory Standards Institute (CLSI). Briefly, a colony of *C. acnes* grown on Schaedler agar plate was inoculated in Schaedler broth and incubated in an anaerobic environment at 37 °C overnight (ON). Then, the inoculum was diluted in 15 mL of fresh medium and incubated until the optical density (OD) at 600 nm of 0.5. The bacterial suspension was diluted to 5 × 10^7^ CFU/mL, and 100 μL was added to each well, obtaining a final density of 2.5 × 10^7^ CFU/well. Meanwhile, *Arctostaphylos uva-ursi* leaf extract was serially diluted in PBS 1X in a concentration range from 10 to 0.07 mg/mL. Vancomycin (10 μg/mL) was used as an antibiotic control (CTRL +) and H_2_O as a solvent control (CTRL −) Subsequently, the plates were incubated at 37 °C in anaerobiosis, and the growth rate was evaluated after 48 h using a microplate reader (Tecan, Männedorf, Switzerland).

### 2.5. Time-Killing Assay

To better understand the *Arctostaphylos uva-ursi* leaf extract kinetics of action in *C. acnes* planktonic cells, the time killing test was performed in accordance with the American Society for Testing and Materials International (ASTM) standard guidelines. Concentrations of 1.25 (2 × MIC), 0.6 (MIC) and 0.3 (½ × MIC) mg/mL, and 2.5 × 10^7^ CFU/mL of bacteria was added to the Schaedler broth in a final volume of 2 mL/tube. Vancomycin (10 μg/mL) and bacteria treated with the solvent used to dissolve the extract (H_2_O) were used as CTRL + and CTRL −, respectively. The bacterial suspension was incubated at 37 °C under orbital shaking (180 rpm) in an anaerobic environment. After the incubation period (0, 3, 6, 9 and 20 h), a 100 μL aliquot of bacterial suspension was serially diluted and plated on Schaedler-agar plates. The plates were incubated at 37 °C for 48 h in anaerobiosis, and the colonies were counted to define CFU/mL [36].

### 2.6. LIVE/DEAD Staining

Bacteria treated with various concentrations of *Arctostaphylos uva-ursi* leaf extract as described in Section 2.3 were examined for cell viability using a BacLight LIVE/DEAD staining kit (Invitrogen, Carlsbad, CA, USA). According to the manufacturer’s instructions, equal volumes of SYTO^®^ 9 and propidium iodide (PI) were mixed and added to each well of the 96-well plate. The samples were incubated for 15 min in the dark at room temperature and observed under a fluorescence microscope. Images were acquired through the Nikon ECLIPSE Ti2-U (Nikon Europe B.V., Amsterdam, The Netherlands) inverted fluorescence microscope with beam settings for FITC, TRITC and merged.

### 2.7. Biofilm Inhibition and Degradation

The ability of *Arctostaphylos uva-ursi* leaf extract to affect biofilm formation and degrade the mature matrix was evaluated using the crystal violet (CV) assay. Briefly, a bacterial inoculum was diluted in a Schaedler broth supplemented with 1% glucose at a final density of 5 × 10^8^ CFU/mL. Regarding biofilm inhibition, a volume of 100 μL of bacterial suspension was transferred to each well of a 96-well plate, and 100 μL of fresh medium supplemented with *Arctostaphylos uva-ursi* leaf extract (10–0.07 mg/mL) was added. The suspension was incubated at 37 °C for 72 h in an anaerobic environment and under static conditions. For the mature biofilm degradation, the bacterial suspension was previously incubated at 37 °C for 72 h in an anaerobic environment and under static conditions. Then, non-adherent cells were removed through two PBS1X washes, and *Arctostaphylos uva-ursi* leaf extract (10–0.07 mg/mL) was used to treat the mature biofilm for 24 h. Biofilms treated with solvent (H_2_O) and treated with vancomycin (20 μg/mL) constituted the CTRL − and CTRL +, respectively. After treatment, residual planktonic cells were removed, and the biofilm was washed twice with PBS1X. Biofilm biomass was quantified by adding 100 μL of 0.01% CV to each well for 30 min at room temperature under orbital shaking. The excess CV was removed and then solubilized with 98% ethanol for 40 min at room temperature under orbital shaking. The absorbance values at 570 nm were obtained using a microplate reader (Tecan, Männedorf, Swiss) [37].

### 2.8. Cytotoxicity and Hemolysis Profile

Cultured Human Keratinocyte (HaCaT) cells were used to evaluate the cytotoxicity of *Arctostaphylos uva-ursi* leaf extract by 3-(4,5-dimethylthiazol-2-yl)-2,5-diphenyltetrazolium bromide (MTT) assay [38,39]. Cells were cultured in DMEM (Thermo Fisher Scientific, Waltham, MA, USA), supplemented with 1% penicillin–streptomycin and 10% fetal bovine serum (Thermo Fisher Scientific, Waltham, MA, USA), at 37 °C with 5% CO_2_ in a humid environment. A density of 2 × 10^4^ cells/well was seeded into 96-well plates and incubated for 24 h. *Arctostaphylos uva-ursi* leaf extract was tested in a concentration range between 10 and 0.07 mg/mL. Cells treated with solvent (H_2_O) and cells treated with 100% DMSO represented CTRL − and CTRL +. After 24 and 48 h of treatment, 100 μL of MTT solution was added to each well for 3 h at 37 °C. Then, the formazan crystals were solubilized by adding 100 μL of 100% DMSO, and the viability rate was recorded at OD at 570 nm, using a microplate reader (Tecan, Männedorf, Switzerland). The *Arctostaphylos uva-ursi* leaf extract haemolytic activity was determined using fresh human erythrocytes from healthy donors [40]. Briefly, 25 mL of blood was centrifuged, and the erythrocytes were washed three times with a NaCl solution (150 mM). Then, the blood was diluted at 1:50 in PBS 1X pH 7.4, and 180 μL of erythrocyte solution was added to the well of a conical bottom 96-well. *Arctostaphylos uva-ursi* leaf extract was added at the same concentrations mentioned above; solvent (H_2_O) and TritonX 0.1% solution represented CTRL − and CTRL +. The samples were incubated under orbital shaking at 37 °C for 60 min and subsequently centrifuged at 500 rpm for 5 min. Haemoglobin release was monitored by measuring the OD of the supernatant at 540 nm.

The selectivity index (SI) of the *Arctostaphylos uva-ursi* was calculated through the ratio between CC_50_ and MIC_50_. Values greater than 3 render the compound rising for in vivo investigation.

### 2.9. Enzyme-linked Immunosorbent Assay (ELISA)

To evaluate the *Arctostaphylos uva-ursi* leaf extract anti-inflammatory proprieties, *C. acnes* fresh colonies were suspended in 5 mL of PBS1X at the final concentration of 5 × 10^8^ CFU/mL. The bacteria suspension was incubated at 80 °C for 30 min, and the heat-killed *C. acnes* was stored at 4 °C until use. Meanwhile, 3 × 10^5^ HaCaT cells were seeded in a 12-well and incubated for 24 h. The following day, cells were stimulated by heat-killed *C. acnes* and (1.2 × 10^7^ CFU/mL, MOI = 20) and 1.25 (2 × MIC), 0.6 (1 × MIC) and 0.3 (½ × MIC) mg/mL of *Arctostaphylos uva-ursi* leaf extract were added. LPS (10 μg/mL) was used as CTRL+, while untreated cells and cells treated with heat-killed *C. acnes*, as an anti-inflammatory (cell) and pro-inflammatory (bact) control. After 24 h, the supernatants of the culture medium were collected for the quantification of the pro-inflammatory cytokine. Levels of IL-8, IL-6, IL-1β and TNF-α were analyzed using a sandwich enzyme-linked immunosorbent assay kit (ELISA) according to the manufacturer’s instructions (Elabscience, Houston, TX, USA). The absorbance was quantified at 490 nm using a microplate spectrophotometer (Tecan, Männedorf, Switzerland).

### 2.10. Antioxidant Activity

The free radical scavenging activity was evaluated by the 2,2-diphenyl-1-picrylhydrazyl (DPPH ∙) assay [41]. Briefly, *Arctostaphylos uva-ursi* leaf extract was serially diluted in a concentration range of 10-0-0.005 mg/mL and mixed with 50 μM of DPPH in methanol. Ascorbic acid (5 mg/mL) and methanol were used as CTRL + and CTRL −. The antioxidant activity was evaluated by reading the absorbance at 517 nm for 15 min and the percentage of Radical Scavenging Activity (RSA) was calculated according to the formula:

RSA (%) = (1 − (Absorbance sample)/(Absorbance control)) × 100

### 2.11. Statistical Analysis

All experiments were performed in triplicate. The data from each experiment represent the mean (SD) of three biological replicates. The ordinary one-way ANOVA, Dunnett’s multiple comparison test and cytotoxic concentration 50% (CC_50_) were performed using GraphPad Prism ver. 8.4.0 for macOS (Software GraphPad, San Diego, CA, USA, www.graphpad.com (accessed on 3 March 2020). Values were considered significant with a *p*-value < 0.05.

## 3. Results

### 3.1. Chemical–Physical Characterization of Arctostaphylos uva-ursi Leaf Extract

**Figure 1:**
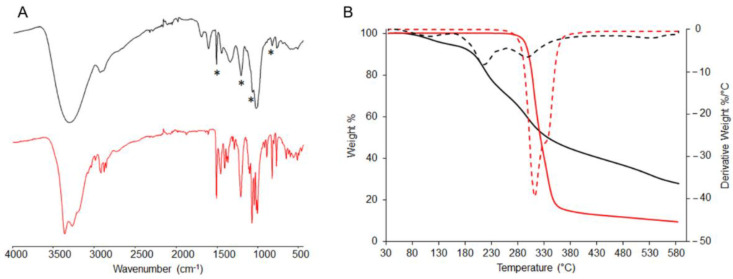
FT-IR/ATR spectra (**A**) and TG/DTG (solid lines/dashed lines) curves (**B**) of *Arctostaphylos uva-ursi* leaf extract (black lines) and arbutin (red lines). In panel (**A**), the asterisks (*) refer to the main arbutin peaks evident in the extract.

In Figure 1A, the FTIR spectra of the *Arctostaphylos uva-ursi* leaf extract and its main component (arbutin) are reported. The peaks at 1509, 1210, 1127 and 830 cm-1 (see asterisks in Figure 1A) can be correlated to the presence of arbutin, containing an aromatic ring. The other peaks could be ascribable to the carbohydrate fraction present in the extract. In Figure 1B, the thermogram (TG) and the derivative thermogram (DTG) traces of the extract and its target molecule, i.e., arbutin, recorded in a nitrogen atmosphere, were reported. Analyzing the extract, a water/volatiles content equal to 5.6%; successively can be observed. The first thermal event, with a peak temperature centered at 215 °C, within the range 160–260 °C, corresponds to a weight loss of 23.8%. Then, a second broadened thermal event was centered at 298 °C, in the range 260–475 °C (29.5% weight loss) and a third centered at 533 °C in the range 477–600 °C (13.2% weight loss). The residue at 600 °C was equal to 27.8%, denoting a high content of a highly stable and/or inorganic fraction. Pure arbutin was also analyzed, evidencing the following features: (i) no water/volatiles content was detected; (ii) two sharps but partially overlapped thermal events fell at 312 and 331 °C, thus indicating that the arbutin decomposition fell in the thermal event ranging in 260–475 °C of the extract; and (iii) a residue at 600 °C equal to 9.3%.

**Figure 2:**
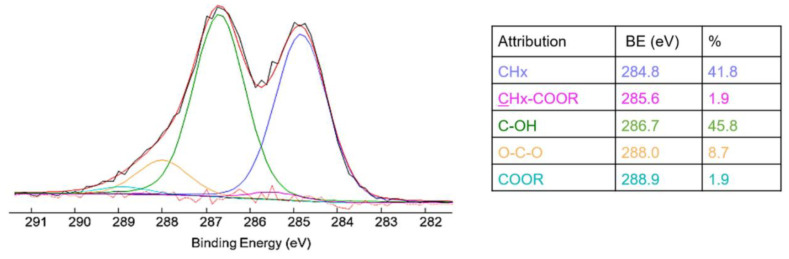
Curve fitting of the high-resolution C1s spectrum and the relevant attributions, BEs values and At%. The uncertainty on the BEs was ±0.2 eV.

XPS analysis of *Arctostaphylos uva-ursi* leaf extract revealed a surface chemical composition based on oxygen (36.2%), carbon (58.5%) and silicon (5.3%). The silicon presence could be probably responsible for the inorganic fraction detected in the extract by TGA. Moreover, in Figure 2 the C1s high-resolution spectrum curve-fitting was reported, evidencing a high C–OH content (45.8% of the total carbonaceous species), indicative of a high abundance of phenolic species in the extract, as well as carbohydrates, (hemi-)celluloses, lignin and other important components (flavonoids, tannins, etc.). Moreover, a small but clearly detectable amount of COOR groups could be related to the presence of some important organic acids, such as ursolic acid, tannic acid, gallic acid, etc. [27]. The C1s curve fitting of arbutin also evidenced a 55.6% contribution of C–OH, as previously reported [34]. The presence of saccharides’ structures, present both in the glycosylated hydroquinone (i.e., arbutin) and in the cellulosic moieties, were additionally evidenced by the detection of hemiacetal groups (O–C–O) at 288.0 eV.

### 3.2. Total Phenol Content (TPC)

The TPC evaluation for the toning lotion based on *Arctostaphylos uva-ursi* extract expressed as mg of gallic acid equivalent GAE/g of the dry extract was equal to 315 ± 15 GAEq/g. The obtained result indicates that the extract employed in this work possesses a total phenol content slightly higher than the *Arctostaphylos uva-ursi* extract TPC values reported in the literature. Indeed, Asensio et al. studied 80 plants sampled in autumn 2014, obtaining TPC values in the range 103–206 GAEq/g and in 2015 in the range 110–201 GAEq/g [29]. Azman et al. found a TPC of about 102 GAEq/g, using 50:50 *v*/*v* EtOH: H_2_O as extraction solvent [27]. Pegg et al. reported a TPC for 95% (*v/v*) ethanol extraction of *Arctostaphylos uva-ursi* of the same order of magnitude as the values we observed (i.e., 312 GAEq/g) [42]. Finally, Sugier et al. reported that the extract from the *Arctostaphylos uva-ursi* leaves collected in the heathlands and in the pine forests exhibited TPC values in the range 285.85–318.28 and 257.51–306.57 GAEq/g, respectively [43].

### 3.3. Franz Cell Permeation Tests

**Figure 3:**
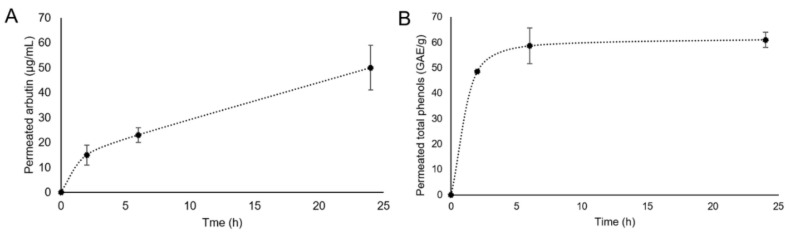
Franz cell permeation kinetics of arbutin (**A**) and total phenols (**B**) detected by HPLC and the Folin–Ciocalteu spectrophotometric method, respectively.

As far as the HPLC analysis, a calibration curve of arbutin standards in the concentration range of 1–100 µg/mL was built up and subsequently HPLC analysis of the extract was carried out by monitoring the arbutin peak. The chromatogram obtained for *Arctostaphylos uva-ursi* extract showed a peak at the retention time of 2.8 min corresponding to arbutin. The toning lotion prepared at a concentration equal to 1.2 mg/mL of the extract, was found to contain 170 ± 20 µg/mL of arbutin. As far as the permeation tests from the toning lotion containing *Arctostaphylos uva-ursi* leaf extract are concerned, the kinetics of arbutin and total phenols permeated in PBS up to 24 h are reported in Figure 3A,B, respectively. It is interesting to highlight that the permeated amount at 24 h represents 29 ± 4% of the total arbutin in the toning lotion. No arbutin adsorbed on the membrane was detected. The total phenol content permeated at 24 h represents 19 ± 1% of the total amount present in the toning lotion. On the other hand, the total phenol content detected on the membrane was found to be equal to 49.7 ± 1.5 GAE/g.

### 3.4. Antibacterial Activity on Planktonic C. acnes

**Figure 4:**
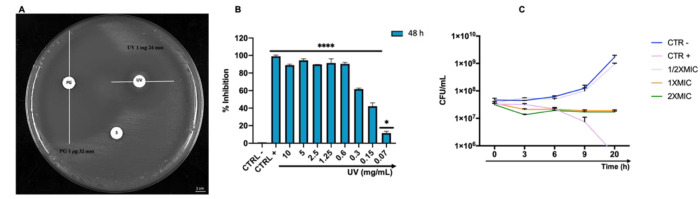
**(A**) Diameter of the inhibition area exhibited by *C. acnes* ATCC 11182 after exposure with *Arctostaphylos uva-ursi* leaf extract (1 mg); piperacillin (1 µg) was used as antibiotic control (PG) while H_2_O as solvent control (S); (**B**) growth inhibition rate (%) of *C. acnes* after *Arctostaphylos uva-ursi* leaf extract treatment; (**C**) time-kill kinetics assay; CTRL −: bacteria treated with the solvent used to dissolve the drug; CTRL +: bacteria treated with vancomycin (10 μg/mL). Data represent the mean ± standard deviation (SD) of three independent experiments; ****: *p*-value< 0.0001; *: *p*-value = 0.0475. ANOVA: *p*-value < 0.0001, R squared = 0.9978.

The antibacterial screening of *Arctostaphylos uva-ursi* leaf extract was evaluated by Kirby–Bauer disk diffusion and broth microdilution assays. Both methods indicated an important antibacterial effect exhibited by the *Arctostaphylos uva-ursi* leaf extract against *C. acnes* ATCC 11827 planktonic cells. The inhibition area obtained from 1 mg of the compound was 24.0 ± 1.2 mm, while no sensitivity was verified following treatment with the solvent (H_2_O) used to dissolve the compound (S). On the other hand, the area of inhibition obtained after treatment with 1 μg Piperacillin (PG) was 32.0 ± 0.9 mm (Figure 4A). To define the MIC value, a dose–response curve was obtained after exposure to *Arctostaphylos uva-ursi* leaf extract in concentrations ranging from 10 to 0.07 mg/mL. The extract showed a performing bacterial activity, exhibiting MIC_50_ and MIC_90_ values of 0.21 and 0.6 mg/mL, respectively. At lower concentrations, a gradual reduction of the inhibitory effect was verified (Figure 4B). The understanding of the *Arctostaphylos uva-ursi* leaf extract kinetic action against *C. acnes* was obtained by time-killing assay. The data obtained showed exponential bacterial growth over time after treatment with the solvent (CTRL −) and with 0.3 mg/mL of *Arctostaphylos uva-ursi* leaf extract (½ × MIC). Exposure to 1.2 (2 × MIC) and 0.6 (1 × MIC) mg/mL maintained the bacterial load constant over time, suggesting a bacteriostatic action. On the other hand, 6 h of exposure to 10 μg/mL of Vancomycin (CTRL +) induced a gradual reduction of the bacterial load, and no living cells were detected after 20 h of treatment (Figure 4C).

### 3.5. Fluorescence Microscopy of Live/Dead Cells

**Figure 5:**
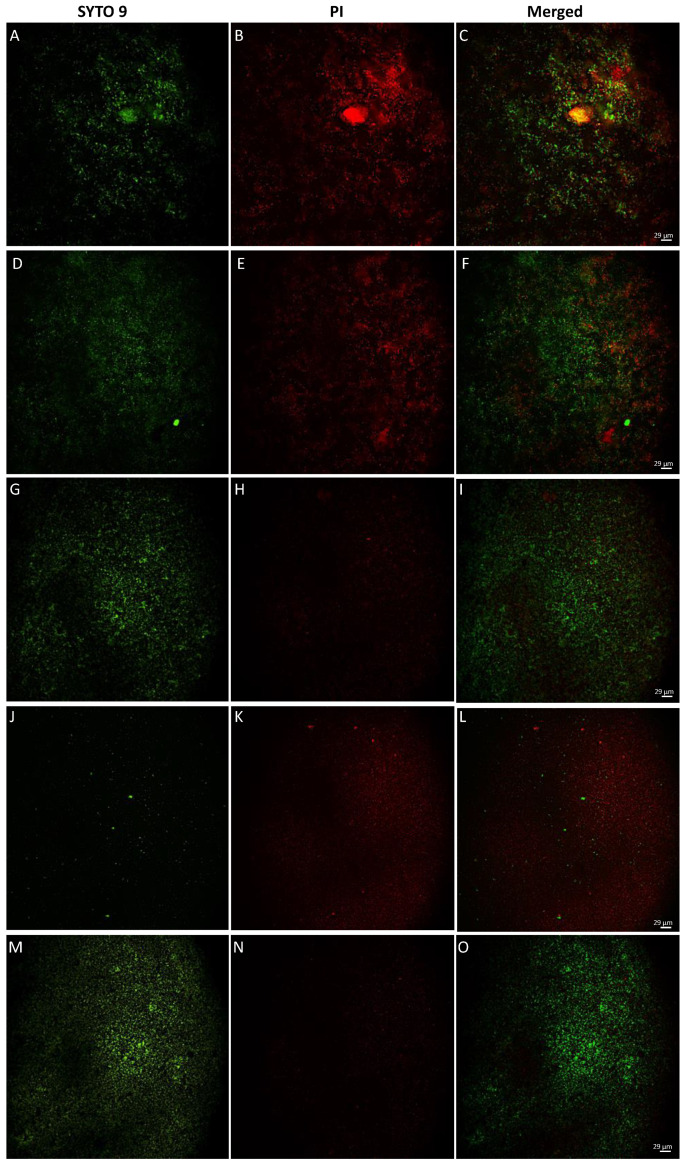
*C. acnes* LIVE/DEAD BacLight staining after treatment with *Arctostaphylos uva-ursi* leaf extract observed under a fluorescence microscope: (**A**–**C**) treatment at 1.2 mg/mL; (**D**–**F**) treatment at 0.6 mg/mL; (**G**–**I**) treatment at 0.3 mg/mL; (**J**–**L**) treatment with vancomycin at 5 μg/mL; (**M**–**O**) untreated bacteria.

To observe the induced bacterial damage, *C. acnes* cells were treated with 1.2 (2 × MIC), 0.6 (1 × MIC), 0.3 (½ × MIC) mg/mL of *Arctostaphylos uva-ursi* leaf extract for 48 h and then observed under fluorescence microscopy after staining with PI and SYTO 9. The images obtained showed two types of staining: (i) *C. acnes* cells with membrane damage in a red fluorescence, which were considered dead; and (ii) cells with intact membrane showing green fluorescence. BacLight LIVE/DEAD results were reported in Figure 5. Cells treated with *Arctostaphylos uva-ursi* leaf extract at 1.2 and 0.6 mg/mL (A–F) showed intense red staining compared to untreated bacteria (M–O), confirming the antibacterial efficacy obtained in previous assays. At the concentration of 0.3 mg/mL, a decrease in cell damage was recorded, increasing the number of live cells stained green (G–I). No viable cells were observed following treatment with vancomycin, which represented the positive control, with bactericidal action (J–L).

### 3.6. Effect of Arctostaphylos uva-ursi on C. acnes Biofilm

**Figure 6:**
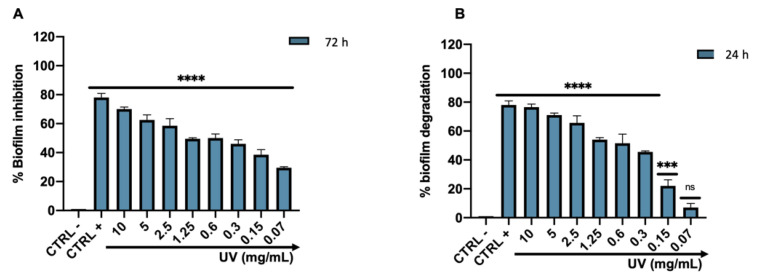
Effect of *Arctostaphylos uva-ursi* leaf extract on the forming (**A**) and mature (**B**) biofilm of *C. acnes* after treatment at the concentration range of 10–0.07 mg/mL. Data represent the mean ± standard deviation (SD) of three independent experiments; **** *p*-value< 0.0001; *** *p*-value = 0.0007; ns *p*-value > 0.05; ANOVA: *p*-value < 0.0001, R squared = 0.9925.

Biofilm formation is one of the main virulence factors of *C. acnes*; therefore the activity of *Arctostaphylos uva-ursi* leaf extract on the forming and preformed biofilm was evaluated. Biofilm biomass was quantified using CV in response to treatment in a concentration range between 10 and 0.07 mg/mL. After 72 h of incubation, *Arctostaphylos uva-ursi* leaf extract inhibited biofilm formation by 73 to 27% at concentrations of 10 to 0.07 mg/mL. In detail, the compound induced inhibition of 50 and 46% compared to the untreated biofilm (CTRL −) at the dose of 0.6 and 0.3 mg/mL, respectively (Figure 6A). On the other hand, the biofilm eradication involved 72 h of mature matrix formation and subsequently 24 h of treatment with *Arctostaphylos uva-ursi* leaf extract in the concentration range mentioned above. Mature biofilm was destroyed in a dose-dependent manner from 78 to 9%, recording an eradication rate of 57 and 45% at 0.6 (1 × MIC) and 0.3 (½ × MIC) mg/mL, respectively. There were no significant changes in the biofilm matrix at concentrations below 0.15 mg/mL (Figure 6B).

### 3.7. Cytotoxicity Profile

**Figure 7:**
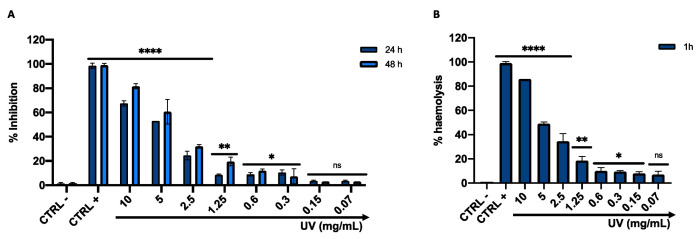
(**A**) HaCaT cytotoxicity after 24 and 48 h of treatment with *Arctostaphylos uva-ursi* leaf extract. (**B**) In vitro hemolysis assay after erythrocyte exposure to *Arctostaphylos uva-ursi* leaf extract. The data represent the mean ± SD. CTRL −: cells treated with the solvent used to dissolve the drug; CTRL+: DMSO used at toxic concentrations (100%) for MTT assay and TritonX 0.1% for hemolysis assay. Dunnett’s multiple comparisons test: **** *p*-value < 0.0001; ** *p*-value = 0.0061; * *p*-value = 0.0301; ns *p*-value > 0.05. Ordinary one-way ANOVA: *p*-value < 0.0001, R squared = 0.9984.

Cell viability was assessed using the HaCaT cell line after 24 and 48 h of *Arctostaphylos uva-ursi* leaf extract exposure in a concentration range between 10 and 0.07 mg/mL. The cytotoxic effect increased in a dose-dependent manner, showing a mortality rate of 50% at 5 mg/mL, after 24 h of treatment. In a dose range between 1.25 and 0.07 mg/mL, *Arctostaphylos uva-ursi* leaf extract did not affect cell viability, recording a live cell rate of more than 80%. No alteration was verified in cellular treatment with the solvent (H_2_O) used to dissolve the compound (CTRL −), while 99% cell mortality was detected by treating HaCaT with 100% DMSO (CTRL +) (Figure 7A). Furthermore, the 50% cytotoxic concentration value (CC_50_), calculated from the dose-effect curves by non-linear regression analysis, was 4.2 mg/mL and 3.6 mg/mL after 24 and 48 h. The calculated SI-value was 17.2, suggesting *Arctostaphylos uva-ursi* as a favorable extract for in vivo investigations. The toxicity profile was confirmed by the hemolysis assay, recording 46 and 35% of erythrocyte lysis after treatment with 5 and 2.5 mg/mL of the compound. At lower concentrations, no relevant hemolysis was detected. (Figure 7B).

### 3.8. Induction of Inflammatory Cytokines by C. acnes In Vitro

**Figure 8:**
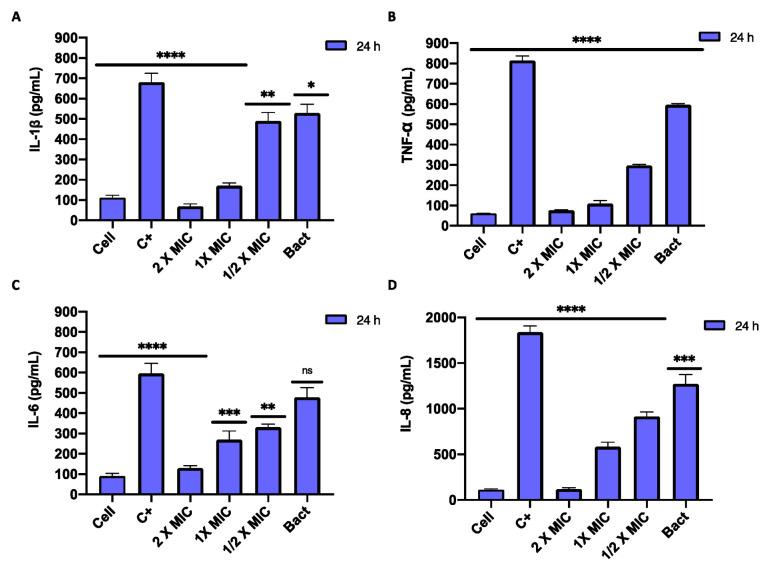
Effects of *Arctostaphylos uva-ursi* leaf extract on pro-inflammatory cytokine production in *C. acnes*-stimulated HaCaT cells. Cells were co-incubated with the compound at 1.2, 0.6 and 0.3 mg/mL and heat-killed *C. acnes,* for 24 h. The cells treated with LPS (10 μg/mL) represent the CTRL+ while untreated cells and cells treated with heat-killed *C. acnes* represent anti-inflammatory (Cell) and pro-inflammatory (Bact) control. The culture supernatants were subsequently isolated and analyzed for the production of IL-1β (**A**); TNF-α (**B**); IL-6 (**C**) and IL-8 (**D**). Data represent the mean ± standard deviation (SD) of three independent experiments; **** *p*-value< 0.0001; *** *p*-value = 0.0004; ** *p*-value = 0.0013; * *p*-value = 0.0311; ns *p*-value > 0.05. ANOVA: *p*-value < 0.0001, R squared = 0.9815.

To investigate the anti-inflammatory effects of *Arctostaphylos uva-ursi* leaf extract in *C. acnes*-treated HaCaT cells, ELISA assays were performed to quantify the pro-inflammatory cytokines. The cell line was incubated with increasing doses of *Arctostaphylos uva-ursi* leaf extract and heat-killed *C. acnes* for 24 h. Treatment with the extract significantly suppressed cytokine production. In detail, cells exposed to *C. acnes* secreted 530, 480, 1300 and 600 pg/mL of IL-1β, IL-6, IL-8 and TNF-α, respectively. On the other hand, treatment with 0.6 mg/mL (1 × MIC) resulted in a decrease in proinflammatory cytokines almost comparable to untreated cells, recording a fold-reduction of 1.8, 1.9, 2.27, 5.4 for IL-1β, IL-6, IL-8 and TNF-α, respectively. At lower concentrations, a slight increase in cytokines was recorded (Figure 8).

### 3.9. Antioxidant Activity

**Figure 9:**
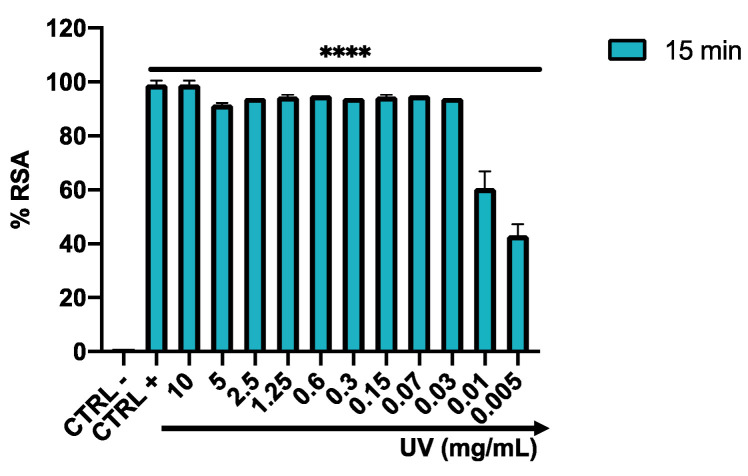
Free radical scavenging activity of *Arctostaphylos uva-ursi* leaf extract. Ascorbic acid (5 mg/mL) and Methanol represent the CTRL + and CTRL −, respectively. DPPH assay represents the mean ± standard deviation (SD) of three independent experiments. **** *p*-value < 0.0001; ANOVA: *p*-value < 0.0001; R squared = 0.9980.

The antioxidant potential of *Arctostaphylos uva-ursi* leaf extract was investigated by evaluating the discoloration of DPPH∙, a free radical that accepts an electron or hydrogen radical to become a stable molecule. Then, the percentage of RSA was calculated in the concentration range from 10 to 0.005 mg/mL. *Arctostaphylos uva-ursi* leaf extract already showed a performing antioxidant power after 15 min, recording radical scavenging effects of over 80% up to 0.03 mg/mL. On the other hand, considering that arbutin contained in 0.03 mg/mL was about 0.006 mg/mL, at this concentration arbutin alone produced a weak radical scavenging activity (i.e., 13%). However, as shown by Takebayashi et al., arbutin may show its radical-scavenging effect in a long-lasting manner and therefore its antioxidant activity, evaluated after 15 min, is underestimated [44]. A linear reduction was observed at concentrations of 0.01 and 0.005 mg/mL, with an RSA rate of 55 and 43%, respectively (Figure 9).

## 4. Discussion

Currently, multi-drug resistance and the side effects associated with the use of conventional drugs require the need to search for new potential sources to fight *C. acnes* infections [45]. Traditional herbal medicines could represent promising approaches to overcome the limitations associated with the use of existing drugs for acne vulgaris treatment [46]. Therefore, the present study evaluated the *Arctostaphylos uva-ursi* leaf-extract ability to fight *C. acnes* in three different properties: (i) antimicrobial potential; (ii) anti-inflammatory properties; and (iii) antioxidant activity.

A preliminary analytical characterization was performed to gain information about the chemical–physical properties of the investigated extract. Arbutin was chosen as the target molecule in the *Arctostaphylos uva-ursi* detection due to its high concentration in the vegetal extract. FT-IR/ATR and XPS analyses clearly evidenced the presence of arbutin, as well as the characteristic spectrum relevant to a complex mixture of molecules/macromolecules of vegetal origin (such as carbohydrates, (hemi-) celluloses, lignin, etc.). Moreover, TGA was used to evaluate the thermal stability of the extract; the detected thermal properties revealed a high thermal stability of the extract (T_onset_ > 150 °C), which makes it suitable for pharmaceutical and/or cosmetic preparations where heating is required.

As far as the Franz cell studies are concerned, the obtained results evidenced that the release and permeation of the different extract compounds followed different pathways. However, it can be concluded that the developed toning lotion carried the *Arctostaphylos uva-ursi* components across the StratM^®^ membrane with a noticeable percentage.

*Arctostaphylos uva-ursi* leaf extract showed a significant inhibitory effect on the growth of *C. acnes*, exhibiting an inhibition area of 24 mm and a bacteriostatic action of up to 0.6 mg/mL. Our results were consistent with the literature data, although differences were found due to growth condition variations and/or bacterial strains investigated. In detail, Hassan et al. reported an inhibition diameter between 15 and 30 mm and an MIC-value between 5 and 1.25 mg/mL against *Escherichia coli (E. coli), Aeromonas caviae*, *Bacillus Cereus*, *Micrococcus luteus*, *Mycobacterium avium* and *Paenibacillus alvei* [47]. The antimicrobial potential could result from polyphenols, which are secreted by plants as a defense mechanism against pathogens. For *Arctostaphylos uva-ursi* leaf extract, it could be associated with the arbutin that represents the main extract component (it makes up 7% to 9% of the leaves) and is already known to exhibit antimicrobial activity [48,49]. Moreover, *Arctostaphylos uva-ursi* was also described to be active against *E. coli* and possess diuretic properties [50,51]. A study on urinary tract infection prevention reported that 18% (27 individuals) and 0% (30 individuals) of women who were given, respectively, placebo and *Arctostaphylos uva-ursi* in combination with dandelion root and leaf developed urinary tract infections [50]. In the second set of experiments, we investigated the action of *Arctostaphylos uva-ursi* leaf extract against the formation and maturation of *C. acnes* biofilm. The CV assay indicated a strong inhibitory and degradative activity on the biofilm matrix. In detail, biomass degradation exceeded 50% and 40% in response to treatment with 1 × MIC and ½ × MIC, thus reaching the deeper layers of a mature biofilm.

In acne inflammation pathogenesis, *C. acnes* plays an important trigger role through IL-6 and IL-8 secretion by follicular keratinocytes and IL-1β, TNF-α, IL-8 and IL-12 by monocytes [52,53]. IL-8, a CXC-type chemokine, is a pro-inflammatory chemotactic factor that primarily affects neutrophils [50]. TNF-α, IL-6 and IL-1β are inflammatory molecules involved in both acute and chronic inflammatory acne [54]. Patients suffering from acne vulgaris presented high levels of IL-8 in peripheral blood mononuclear cells and remarkable expression of IL-6 and IL-8 in keratinocytes [55]. Since these mediators aggravate the initial acne lesion by increasing the inflammatory state, we evaluated whether *Arctostaphylos uva-ursi* leaf extract could inhibit the secretion of the pro-inflammatory cytokines in HaCaT cells infected with *C. acnes*. Our evidence showed strong induction of *C. acnes* to the secretion of IL-1β, TNF-α, IL-8 and IL-6 in keratinocytes [56]. In co-presence with *Arctostaphylos uva-ursi* leaf extract bacterium, a marked cytokine suppressive effect was recorded, demonstrating a strong anti-inflammatory effect associated with the antibacterial properties mentioned above. Consistent with our findings, a study by Weber et al. screened ninety-nine herbal extracts by evaluating their TLR2- and TLR4-dependent anti-inflammatory effects in THP-1 monocytes. Among these, *Arctostaphylos uva-ursi* leaves, *Castanea sativa* leaves, *Cinchona pubescens* bark, *Cinnamomum verum* bark, *Salix alba* bark, *Rheum palmatum* root, *Alchemilla vulgaris* plant, *Humulus lupulus* cones, *Vaccinium myrtillus* berries and *Curcuma longa* root showed the highest anti-inflammatory properties, suppressing the production of proinflammatory cytokines. In detail, *Arctostaphylos uva-ursi* inhibited LPS-induced NF-κB translocation, suppressed TNF-α secretion and induced IL-10 release in macrophages, indicating a transition from M1 macrophages (pro-inflammatory) to M2 macrophages (anti-inflammatory) [57]. The predominant molecule responsible for the antioxidant anti-inflammatory activity is arbutin, which reduces the secretion of IL-1β and TNF-α and other related genes, such as the chemotactic protein monocyte-1 and IL-6 [58]. Finally, considering the growing interest in “green production” and in research of natural antioxidants, the radical scavenging activity of *Arctostaphylos uva-ursi* leaf extract was determined by DPPH assay. The high percentage of RSA obtained up to a concentration of 0.15 mg/mL indicated a performing antioxidant potential, attributable to the high phenol content in *Arctostaphylos uva-ursi* leaves, as confirmed by the high levels of phenolic compounds detected in the extract (315 GAEq/g).

Considering this information, *Arctostaphylos uva-ursi* can be considered a valuable plant source for the pharmaceutical and cosmetic fields. Considering that *C. acnes* in recent years has shown a worrying increase in resistance to erythromycin, clindamycin, doxycycline, tetracycline, trimethoprim/sulfamethoxazole, levofloxacin and minocycline [59], the discovery of green alternative sources as antimicrobial and anti-inflammatory agents for the Acne vulgaris treatment could overcome the limitations associated with *C. acnes* multidrug resistance and mitigate the side effects of conventional medications. Furthermore, the high phenol content could make *Arctostaphylos uva-ursi* a promising natural antioxidant additive in herbal preparations. Nevertheless, further investigation to better understand the *Arctostaphylos uva-ursi* mechanism of action against *C. acnes* and to designate additional inflammatory targets will be necessary.

## Data Availability

The data presented in this study are available herein.
